# Supplementary material for: Experimental, Theoretical and Numerical Studies on Thermal Properties of Lightweight 3D Printed Graphene-Based Discs with Designed Ad Hoc Air Cavities
Source: Nanomaterials (Basel). 2023 Jun 15;13(12):1863. doi: 10.3390/nano13121863 (PMC10302392; doi:10.3390/nano13121863)
Supplement: Supplementary file 1 [file nanomaterials-13-01863-s001.zip › nanomaterials-2456668-supplementary.pdf]

nanomaterials

Supplementary Materials

Experimental, Theoretical and Numerical Studies on Thermal Properties of Lightweight 3D-printed Graphene-based Discs with Designed Ad-hoc Air-cavities

Giovanni Spinelli [1,2]*, Rosella Guarini[2], Rumiana Kotsilkova[2], Evgeni Ivanov[2,3], and Vittorio Romano[4]

[1]University of Study "Giustino Fortunato", Via Raffaele Delcogliano 12, 82100, Benevento, Italy;
[2]Institute of Mechanics, Bulgarian Academy of Sciences, Acad. G. Bonchev Str., Block 4, 1113, Sofia, Bulgaria; rgrosagi@gmail.com (R.G.); kotsilkova@yahoo.com (R.K.); ivanov_evgeni@yahoo.com (E.I.);
[3] Research and Development of Nanomaterials and Nanotechnologies (NanoTech Lab Ltd.), Acad. G. Bonchev Str., Block 4, 1113, Sofia, Bulgaria
[4] Department of Industrial Engineering, University of Salerno, Via Giovanni Paolo II, 84084 Fisciano (SA) Italy; vromano@unisa.it (V.R.);
* Correspondence: spinelligiovanni76@gmail.com; Tel.: +359 2 979 6476

Citation: Lastname, F.; Lastname, F.; Lastname, F. Title. *Nanomaterials* **2023**, *13*, x. https://doi.org/10.3390/xxxxx

Academic Editor: Firstname Lastname

Received: date
Accepted: date
Published: date

Publisher's Note: MDPI stays neutral with regard to jurisdictional claims in published maps and institutional affiliations.

Copyright: © 2023 by the authors. Submitted for possible open access publication under the terms and conditions of the Creative Commons Attribution (CC BY) license (https://creativecommons.org/licenses/by/4.0/).

2. Materials and Methods

The production line from the beginning of the filament extrusion up to 3D-printed samples is schematically illustrated in Figure S1, whereas the main selected printing parameters are reported in Table S1.

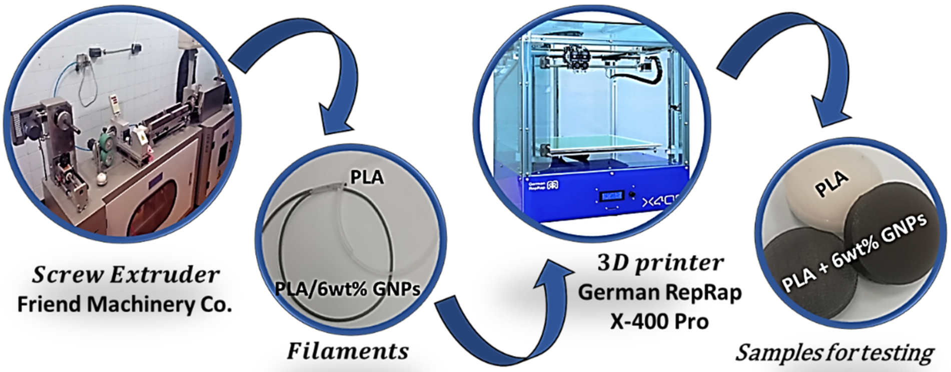

Figure S1. Production line of the test samples. From left to right: screw extruder for filaments manufacturing to be used with a 3D printer in order to obtain specimens for testing.

Table S1. Printing parameters setting.

Parameter	Value	Unit
Nozzle temperature	210-220	°C
Nozzle Diameter	0.4 (PLA) – 0.5 (PLA with filler)	mm
Bed temperature	65	°C
Extrusion speed	17	mm/s
Extrusion multiplier	0.8	-
Extrusion width	0.4	mm
Primary layer height	0.25	mm
Internal infill pattern	Rectangular	-
External infill pattern	Rectangular	-
Retraction length (distance)	1	mm
Retraction speed	30	mm/s
Infill density	100	%
Cooling rate	100	%

LFA is based on the physical principle summarized in the schematic representation of Figure S2. One side of a plane-parallel sample is heated through a proper light pulse (with a Gaussian distribution in time) and the consequent increases in temperature at the sample's opposite face is tracked over time. Such temperature rise is strictly closed to the thermal diffusivity of material: the higher it is, the faster the temperature propagates toward the backside.

The pulse is selected in duration and intensity such as to heat up, as uniform as possible, the opposite face of sample to around 1 K. Once the time value at half temperature course (half time $t_{1/2}$, as shown in Figure 3) is determined and knowing the sample thickness (*Δz*), the thermal diffusivity (α) is calculated in agreement with the following equation [39]:

$$\alpha = 0.1388 \cdot \frac{\Delta z^2}{t_{1/2}} \quad \text{(S1)}$$

and then the thermal conductivity (λ) as follows:

$$\lambda(T) = \alpha(T) \cdot \rho(T) \cdot c_p(T) \quad \text{(S2)}$$

where: $\rho(T)$ represent the density [kg/m^3] and $c_p(T)$ is the specific heat [J/kg·K] of the material, which value is determined by a comparison between the height (ΔTmax) observed for the test specimen with that of a reference material.

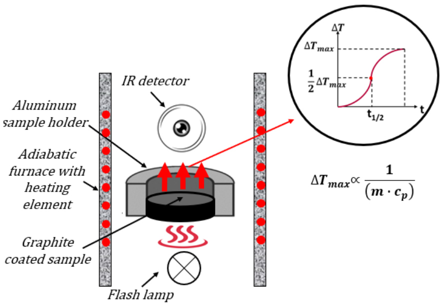

Figure S2. Physical principle of a LFA with key parameters for the determination of the thermophysical properties.

The heat transfers inside the body from the heated surface to the opposite face by conduction, according to the Fourier's equation:

$$q = -\lambda \cdot \nabla T \quad \text{(S3)}$$

where:

- q is the heat flux, [W/m^2];
- λ is the thermal conductivity of the involved material, [W/m·K];
- ∇T is the temperature gradient [K/m].

In the Cartesian coordinate system, the thermal energy equation to describe the conductive transport at constant pressure on a differential volume Δx·Δy·Δz, can be written as follows [38]:

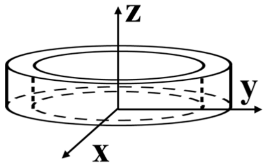

$$-\left[\frac{\partial}{\partial x}\left(\frac{\partial q_x}{\partial x}\right) + \frac{\partial}{\partial x}\left(\frac{\partial q_x}{\partial x}\right) + \frac{\partial}{\partial z}\left(\frac{\partial q_z}{\partial z}\right)\right] = \rho c_p \frac{\partial T}{\partial t} \quad \text{(S4)}$$

where:

- ρ is the density of the material [kg/m^3];
- c_p is the specific heat of material [J/kg·K];
- λ is the thermal conductivity of the involved material, [W/m·K]
- q_x, q_y and q_z [W/m^2] are the conductive heat flux components along x, y and z directions, expressed by Fourier's law according to the following relations:

$$q_x = -\lambda \frac{\partial T}{\partial x} \qquad q_y = -\lambda \frac{\partial T}{\partial y} \qquad q_z = -\lambda \frac{\partial T}{\partial z} \tag{S5}$$

The minus sign which appears in all flow components is due when in the positive direction of the thermal flow the temperature gradient is negative.

Substituting the expressions of the conductive fluxes q_x, q_y and q_z, the balance equation becomes:

$$\frac{\partial}{\partial x}\left(\lambda \frac{\partial T}{\partial x}\right) + \frac{\partial}{\partial y}\left(\lambda \frac{\partial T}{\partial y}\right) + \frac{\partial}{\partial z}\left(\lambda \frac{\partial T}{\partial z}\right) = \rho c_p \frac{\partial T}{\partial t} \tag{S6}$$
